# Immunotherapy-Related Oral Adverse Effects: Immediate Sequelae, Chronicity and Secondary Cancer

**DOI:** 10.3390/cancers15194781

**Published:** 2023-09-28

**Authors:** Sharon Elad, Noam Yarom, Yehuda Zadik

**Affiliations:** 1 Oral Medicine, Eastman Institute for Oral Health, University of Rochester Medical Center, Rochester, NY 14620, USA; 2 Oral Medicine Unit, Sheba Medical Center, Tel Hashomer 5265601, Israel; 3 School of Dental Medicine, Tel Aviv University, Tel Aviv 6997801, Israel; 4 Department of Oral Medicine, Sedation and Imaging, Faculty of Dental Medicine, Hebrew University of Jerusalem, Hadassah Medical Center, Jerusalem 9112001, Israel; yehuda.zadik@mail.huji.ac.il; 5 Department of Military Medicine, Faculty of Medicine, Hebrew University of Jerusalem, Jerusalem 9112001, Israel

**Keywords:** adverse effect, cancer, immunotherapy, lichen planus, mucosa, oral, salivary gland, Sjögren syndrome

## Abstract

**Simple Summary:**

This paper characterizes the immunotherapy-related adverse effects in the oral tissues, in a large series of patients. This includes a description of the severity of oral symptoms, immediate clinical presentation, treatment, chronicity despite holding the immunotherapy, and the development of oral cancer. The management of these patients exemplified new diagnostic tools, detailed clinical presentation that may assist to differentiate between various oral irAEs and outlines unique adjustments in the topical treatment and inclusion of a new treatment modality for these irAEs. Lastly, this paper raises the awareness for second primary oral cancer and possible risk for oral malignant transformation.

**Abstract:**

(1) Background: Immunotherapy-related adverse effects (irAEs) have been reported to manifest in oral tissues, mainly as lichenoid and non-lichenoid lesions and salivary gland dysfunction; however, the characterization of oral irAEs and their clinical impact is limited. (2) Methods: This is a retrospective clinical chart review of 14 patients with oral irAEs, describing the impact of the oral irAEs in terms of the immediate effect, treatment, chronicity of the irAEs and the development of oral cancer. (3) Results: Common symptoms were pain and dry mouth, causing no-to-severe pain and/or dry mouth sensation. The immediate sequala ranged from sensitivity to certain foods up to elimination of oral intake. Treatment included conventional palliation techniques with or without systemic steroids. Discontinuation of the immunotherapy agents was required in 6 patients. Innovative treatment modalities included photobiomodulation for oral mucosal pain relief, and salivary gland intraductal irrigations for relief of salivary gland hypofunction. Late sequala included the development of proliferative leukoplakia and oral cancer. (4) Conclusions: Patients treated with immunotherapy may develop debilitating oral irAEs. They should be followed for oral involvement so treatment may be initiated when the symptoms are mild to avoid discontinuation of the immunotherapy. Patients that develop oral lichenoid lesions should receive long-term follow-up, as they may have higher risk for oral cancer.

## 1. Introduction

Immunotherapy is an innovative concept in cancer therapy that has recently become the standard of care in certain malignancies [1]. Immunotherapy agents cause the release of physiological inhibitory mechanisms that are intended to prevent the unwanted immune attack of normal cells. By unblocking immune checkpoints, the cytotoxic T-cell-mediated immune system is able to attack and destroy cancer cells.

As the use of FDA-approved immunotherapy drugs increases, our understanding of the treatment outcomes improves. Specifically, there are data about the following agents: anti-PD-1 checkpoint inhibitors (nivolumab, pembrolizumab, cemiplimab-rwlc, dostarlimab-gxly, and nivolumab and relatlimab-rmbw), anti-cytotoxic T-lymphocyte antigen-4 (CTLA-4) checkpoint inhibitors (ipilimumab), anti-programmed death-ligand 1 (PD-L1) checkpoint inhibitors (atezolizumab, avelumab, and durvalumab), anti-vascular endothelial growth factor (VEGFR)2 checkpoint inhibitors (ramucirumab), lymphocyte-actiation gene 3 (LAG-3) inhibitors (relatlimab, nivolumab and relatlimab-rmbw), and CD3 targeted bispecific antibodies (teclistamab, blinatumomab). Additional drugs are being developed.

Although these agents are beneficial in terms of overall survival and cancer progress-free survival, some immunotherapy-related adverse effects (irAEs) have been reported [1]. The common systems involved are skin, endocrine organs, gastrointestinal tract and liver [2].

IrAEs are prevalent, affecting up to 90% and 70% of patients treated with anti-CTLA-4 and anti-PD-1/PD-L1, respectively [3]. The incidence of grade 3/4 irAEs varies greatly, ranging between 0–66% [4]. The incidence of irAEs is higher in immunotherapy combination protocols compared to a single-immunotherapy agent [5,6].

The oral irAEs reportedly involve the oral mucosa and salivary glands [7,8], manifesting mainly as lichenoid and non-lichenoid lesions and salivary gland dysfunction, respectively [9,10,11]. Dysgeusia is reported sparsely, as well as anecdotal reports of medication-related osteonecrosis of the jaw and oral burning sensation [12]. Oral irAEs, as part of a generalized erythema multiforme or Steven Johnson syndrome, have been reported too [7]. The impact of these oral irAEs ranges from minor discomfort to severe excruciating pain. Immunotherapy has often been discontinued because of oral irAEs [1,13].

The frequency of other oral mucosal irAEs are poorly understood, as the evidence is mostly based on large phase III studies which are typically not focused on the specific oral manifestations. Likewise, there is some inconsistency in the reporting and terminology of oral mucosal presentation, which makes it challenging to assess the frequency of each oral entity. Of note, in a single center study, the overall frequency of oral irAEs was 6.8% (317 of 4683 patients) and the incidence rates for oral mucosal disorder and dysgeusia were 2.3%, and 1.6%, respectively [14]. Regarding the frequency of salivary glands involvement in irAEs, the literature shows a range of 0.3–9.4% [14,15].

Detailed reports of oral irAEs are scarce. This lack of coverage may cause misunderstandings about the types of oral manifestations, diagnostic tests needed, and even incorrect expectations about treatment outcomes. Therefore, the aim of this paper is to present an in-depth description of a relatively large group of patients with oral irAEs and discuss the implications of these adverse effects.

## 2. Methods

A retrospective chart review was conducted, of a cohort of cancer patients who were treated with immunotherapy and developed oral irAEs. The patients’ medical comorbidities, history of present illness, clinical presentation, pertinent histopathologic and imaging findings, and disease management were studied. The impact of the irAEs in terms of immediate effect on the ongoing cancer therapy, severity of symptoms, chronicity of the irAEs, and development of oral cancer were assessed. This paper does not include patients who were described by us in previous published works [9,10].

## 3. Results

Table 1 presents the demographics of 14 patients with oral irAEs who were studied (10 male, 4 female), with a median age of 62.5 years old (range 33–83). The majority of the patients were diagnosed with either a melanoma, head and neck cancer, or lung cancer, and all except one had metastasis. Common co-morbidities were hypertension, diabetes mellitus, gastroesophageal reflux disease, benign prostatic hyperplasia, and thyroid disease. Common medications included metformin, prednisone, and zoledronic acid. Cancer treatments included surgery, chemotherapy, and radiotherapy, with 3 patients receiving radiotherapy to the head or neck. All of the patients received immunotherapy with either pembrolizumab (*n* = 7), nivolumab (*n* = 3), or a combination of nivolumab and ipilimumab (*n* = 4). In 2 patients there was concurrent treatment with lenvatinib.

Table 2 presents the oral mucosal involvement with oral irAEs. Most of the patients reported severe pain (5–10) and/or severe sensitivity (7–10), which had a great impact on their ability to eat. Two patients (C2, A1) reported weight loss of 26–30 pounds due to pain/burning in the mouth and difficulty eating. Discontinuation of the immunotherapy agents was required in 6 of the 14 patients. Symptoms included pain, burning, and dysphagia, as well as swelling and bleeding of the mouth and lips. Symptoms were aggravated by salty or acidic food. The clinical presentation included primarily lichenoid lesions, erosive lesions, and ulcers. These were mostly observed in the buccal mucosa, labial mucosa, lips, and gingivae, but multiple cases also had irAE involvement in the tongue and hard palate. The gums presented either desquamative gingivitis, white striae, or a generalized purulent gingival enlargement.

**Figure 1:**
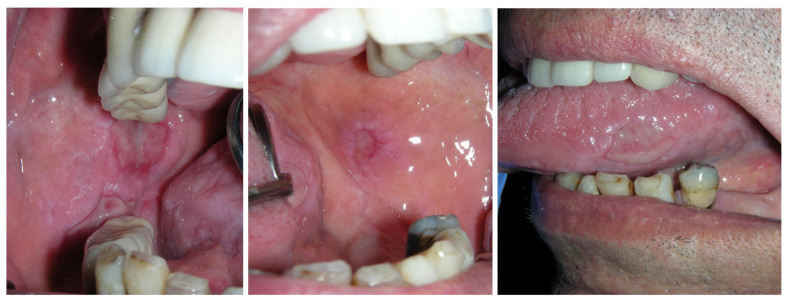
Lichen planus pemphigoides (A4).

Biopsies showed a variety of results, including lichenoid features, subepithelial split, lichen planus pemphigoides (Figure 1) peripheral giant cell granuloma, and non-specific inflammation and ulceration. Direct immunofluorescence was only available for five patients, showing inconsistent results. Two patients were negative for all staining (A2, C2), whereas one patient (A4) presented intercellular staining for IgM and C3, another patient (A7) presented staining for linear fibrinogen at the basement membrane zone, and the last patient presented strong junctional staining for IgG, and C3 (B1). The correlation between the clinical, histopathologic, and immunofluorescent findings were necessary to interpret the diagnosis, particularly in light of the mixed diagnoses.

**Figure 2:**
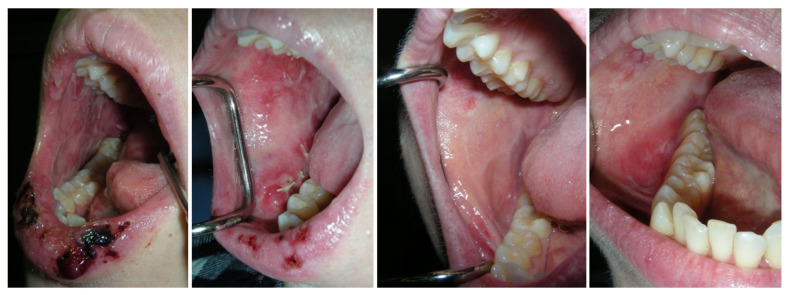
Lichenoid lesions and lip crusts in a patient treated for melanoma with a single dose of Nivolumab + Ipilimumab, and then with another 4 doses of Nivolumab (A3). There was prompt, complete resolution of the oral lesions with systemic and topical steroids. The lesion recurred 2 months following discontinuation of the immunotherapy.

Table 3 presents treatments for mucosal irAEs. The majority of patients needed to stop immunotherapy due to oral irAEs, and many were prescribed systemic steroids (prednisone with a starting dose of 40–60 mg/d). Topical steroids were also used in most of the cases, and in higher concentrations than a typical first-line treatment. Treatments also included antifungal (nystatin), antimicrobial (chlorhexidine), and moistening agents. Most of the patients responded well to these treatments; however, the oral lesions were not necessarily resolved. In 10 out of the 12 patients with oral mucosal irAEs, the oral mucosal disease persisted and turned into a chronic low-grade oral disease. Furthermore, this report describes a flare-up of oral lichenoid lesions 2 months after the immunotherapy was held, after which the oral irAEs seemed to subside (A3) (Figure 2).

**Figure 3:**
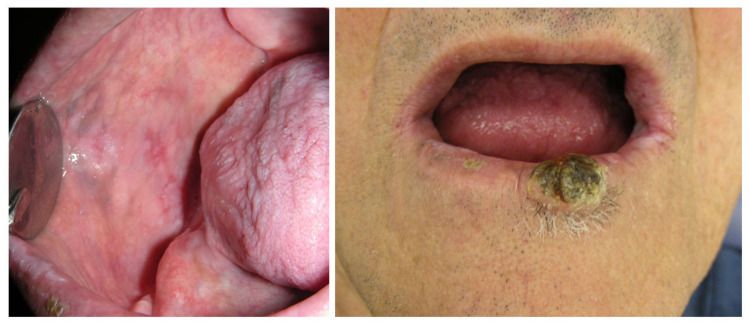
Well-differentiated squamous cell cancer of the lip developed in a patient treated with nivolumab for adenocarcinoma of the lung (A7). Intra-oral presentation includes bilateral mixed white-red lesions with a lichenoid appearance. Microscopic evaluation of a tissue specimen taken from the right buccal mucosa identified lichenoid reaction.

Two patients developed oral cancer while treated with immunotherapy. One patient developed squamous cell carcinoma of the tongue while being treated with pembrolizumab for nasopharyngeal carcinoma (A4). Another patient developed lip squamous cell carcinoma while he was treated with nivolumab for adenocarcinoma of the lung (A7) (Figure 3). These were considered second primary cancers and not metastasis. Another patient developed proliferative leukoplakia (PL) arising from oral lichenoid lesions within 7 months of diagnosis of oral lichenoid, and within 12 months of nivolumab administration (C2).

**Figure 4:**
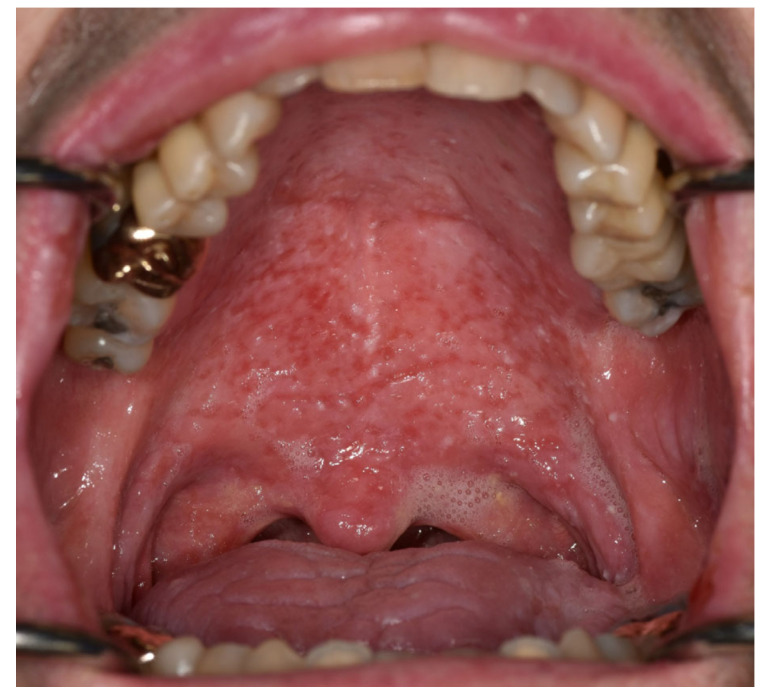
Deep pin-sized ulcers at the distribution of the minor salivary glands with generalized erythema of the remaining surface (C1). Mucoid saliva deposits adhere to the oral surface, indicating poor saliva flow.

Table 4 presents the salivary gland irAEs. Salivary gland symptoms included xerostomia, dysphagia, difficulty sleeping, loss of appetite, and difficulty speaking. These symptoms were worsened by mouth breathing, talking, and eating. Four out of the eight patients with salivary gland irAEs presented clinically with thick, sticky, frothy saliva. Additional common signs related to salivary gland irAEs included a glossy appearance of the oral mucosa, and dry and cracked lips. The two patients with sialometry measurements showed normal total volume; however, there was a high proportion of serous to mucous fractions (0.5:0.5 mL in one patient [A1] and 0.3:2.7 mL in another patient [C1]). The minor salivary gland involvement presented as mild-to-severe erythema in a multi-punctuate palatal pattern. In its most severe form, the presentation included multiple pin-sized ulcers in the distribution of the minor salivary gland, which coalesced to continuous deep erosive lesions (Figure 4). These lesions healed slowly despite systemic and topical steroids. Three patients presented with diffuse erythema in the palate, which was interpreted as oral candidiasis. Cone-beam computerized tomography (CBCT) sialogram was performed for one patient (A5) indicating slow emptying of the parotid gland.

Table 5 presents treatments for salivary gland irAEs. Approximately half of the patients needed to stop immunotherapy due to salivary gland irAEs. Some patients were prescribed systemic steroids (prednisone with a starting dose of 15–60 mg/d) or pilocarpine. Other treatments for dry mouth included moistening agents (liquid or adhesive dissolving discs), sipping water, gustatory stimulation, acupuncture, and salivary gland intraductal irrigation. Most of the patients responded gradually and only partially to treatment. Topical antifungal (nystatin) was prescribed when co-diagnosis of oral candidiasis was suspected.

## 4. Discussion

This report presents the marked impact of oral irAEs on patients’ well-being. The common oral mucosal irAEs were lichenoid, erosive, and ulcerative lesions, and they developed within 2–12 months following the initiation of immunotherapy. Most patients reported severe oral pain or burning sensation at the onset of the oral irAEs. This led to a hold on immunotherapy in about half of the patients. The initial response to the hold on immunotherapy and high-dose steroids was good; however, most patients experienced symptoms and presented with oral irAEs months following the discontinuation of the immunotherapy agents, and flare-up was observed even if the initial response to treatment seemed to be complete. These findings concur with the nature of oral lichen planus and oral lichenoid lesions, which are known to be chronic disorders. In 2 of our patients, the oral irAEs were severe, eliminating any oral intake (A1, C2). In oral lichenoid leasions that are associated with medications, holding the suspected offending agent does not guarantee reversal of the oral lesions or discontinuation of the oral lesions [16]. Thus, these patients need long-term care, corresponding with the guidelines for the management of oral lichen planus and oral lichenoid lesions [16,17].

The report presents a series of histopathologic and immunofluorescence findings, which support the diagnosis of oral lichen planus or oral lichenoid lesions. However, some of the findings were unique, including lichen planus pemphigoides (A4) and pemphigoid with coexistence of clinical presentation compatible with lichenoid (B1). Similarly, to our case, the literature reports the coexistence of oral mucous membrane pemphigoid and clinical lichenoid drug reaction in a patient treated with toripalimab and pembrolizumab [18]. These cases raise the question of whether exposure to the anti-PD-1 drugs attacks a single molecular target that is common to the pathogenesis of both pemphigoid and lichen planus, or if the initial pemphigoid cascades into lichenoid presentation. Alternatively, the combination of clinical and microscopic features of pemphigoid and lichenoid may represent paraneoplastic autoimmune multiorgan syndrome (PAMS). Our findings highlight the importance of oral biopsy in irAEs, especially when the oral presentation is non-diagnostic erosions or ulcers. Once a diagnosis of oral pemphigoid or its variants is made, it is advised to refer the patient to an ophthalmologist to assess for conjunctival involvement.

Salivary gland involvement was associated with severe symptoms. Beyond severe dry mouth symptoms, patients had difficulty swallowing, speaking, and sleeping. Indirectly, these affected the patients’ ability to work (teaching) or socialize. The clinical presentation was not necessarily hyposalivation, but rather increased mucoid fraction of the saliva, both clinically and on sialometry. The observation of normal volume but altered quality of saliva also corresponds with the imaging finding in one of our patients, in which CBCT-sialogram showed normal morphology but reduced function [19]. The literature reports thick saliva salivary gland hypofunction in a series of 18 patients treated with immunotherapy agents (nivolumab, pembrolizumab, and avelumab) [13]. In this report, most of the patients scored the symptoms as grade 2 on the Common Terminology Criteria for Adverse Events (CTCAE) ver. 5.0 scale, meaning ‘moderate symptoms; oral intake alterations (e.g., copious water, other lubricants, diet limited to purees and/or soft, moist foods); unstimulated saliva 0.1–0.2 mL/min’ [20]. This clinical description corresponds with the findings in our group of patients; however, the unstimulated whole salivary flow was not necessarily low, but rather indicated an increased mucoid fraction. Therefore, it is unclear if the CTCAE scale for salivary gland toxicity would provide a good indication for the salivary gland irAEs.

The effect of the dry mouth was noticed on the oral mucosa and lips, including glossy appearance of the oral surface, tongue depapillation, cracked lips, and development of oral candidiasis. Injury to the minor salivary glands also seemed to be associated with oral mucosal lesions, presenting as generalized multi-punctuate erythema or erosions. These lesions healed relatively slowly despite systemic and topical steroids, lagging behind the symptomatic improvement in dry mouth sensation.

The literature presents a similarity between the histopathology of the minor salivary gland in immune-check inhibitors and Sjögren syndrome, with sialoadenitis and lymphocytic infiltrate [13,21]. However, the characterization of the infiltrate differed from Sjögren, in both its wider distribution within the tissue and its cellular diversity [21]. Furthermore, only a minority of the patients reported the typical serology observed in patients with Sjögren syndrome [13,21]. This suggests that the pathogenesis of the salivary gland irAEs is different than in Sjögren syndrome.

Radiotherapy to the head and neck was delivered in some of the patients and may have increased the symptoms of dry mouth. However, in most of these patients, the radiotherapy was carried out long prior to the administration of immunotherapy; thus, the rapid increase in dry mouth symptoms is attributable to the addition of immunotherapy. Additionally, co-administration of targeted therapy, for which the effect on salivary gland function is unclear, may confounded the patients’ symptoms or the clinical findings. Clearly, with multiple cancer therapies typically delivered simultaneously, isolation of the effect of immunotherapy on the salivary gland is not feasible in a relatively small cohort.

One of the biopsies diagnosed a peripheral giant cell granuloma arising in an oral lichenoid reaction (B3). It was sampled from an exophytic lesion in a patient with generalized lichenoid gingival presentation. Both lichenoid and chronic granulomatous reaction are mediated by chronic inflammation [22,23]. Lichenoid granulomatous reactions were reported in the oral tissues, more commonly with solitary lesions involving the gingival, buccal, and vestibular mucosa [22]. Their clinical presentation varies with a differential diagnosis including oral lichen planus, vesiculobullous lesions, leukoplakia, dysplasia, and squamous cell carcinoma. This non-specific clinical presentation highlights the importance of a biopsy from oral lesions in patients treated with immunotherapy.

This paper presents 2 cases of oral malignancy that developed in patients while being treated with pembrolizumab or nivolumab (A4, A7). Prior to this complication, oral lichenoid erosive and ulcerative lesions were observed, which raises the question about the potential for malignant transformation of irAEs oral lichenoid lesions. Of note, while drug-related lichenoid reaction is not considered to be a potentially malignant disorder, oral lichenoid as a manifestation of an autoimmune disease may pose a higher risk for oral malignant transformation [24]. This report also presents a case of nivolumab-related oral lichenoid lesions, which during the 7-month follow-up period on the oral irAEs turned into PL. The World Health Organization (WHO) considers oral lichen planus to be a potential malignant disorder, with estimated risk of 1.09% [17,25]. The data about oral lichenoid reaction are more controversial. Likewise, the literature suggests high malignant transformation risk in proliferative verrucous leukoplakia [25,26]. These cases suggest that patients who develop oral mucosal irAEs should be followed up on regularly to surveil for malignant transformation. The frequency of the follow-up may vary based on the type of oral mucosal lesions and should typically be delivered once or twice a year. For more irregular patterns of oral lesions that are observed, oral exams should be more frequent.

The interaction of the immune check inhibitors (ICI) with the PD-1, PD-L1, and CTLA-4 in the tissues leads to a disruption of the host T-cell signaling and the upregulation of the T-cell immune response against cancer cells. While this effect helps to fight the cancer cells, it also affects normal tissues. It was hypothesized that the irAEs may be a result of increased T-cell proliferation or decreased Treg-mediated immunosuppression that is attributed to anti-CTLA-4. Likewise, it was speculated that activation of certain T-cell clones by anti-PD-1 may lead to irAEs [27,28]. Information on the pathogenesis of oral irAEs is very limited, and the understanding is based mostly on extrapolation from knowledge on equivalent oral diseases. For example, the above-mentioned proposed mechanisms for irAEs conform with the understanding about the pathogenesis of oral lichen planus (OLP). In OLP, chronic dysregulation of the immune response to the offending antigens lead to increased cytokines and increased adhesion molecule expression. These in turn recruit T-cells and mast cells as part of a complex interplay between various cells, such as CD8+ cytotoxic cells, CD4+ Th1 T-cells, as well as involvement from Th9, Th17, and Treg T-cells [29].

The range of treatments for the oral mucosal disease was adjusted to the severity of the oral irAEs. Generally, systemic steroids are used when multiple systems are affected by irAEs, whereas topical steroids are used to enhance the treatment topically or when the oral mucosa is the only site of irAEs. The topical steroids commonly prescribed are dexamethasone 0.02–0.05% rinse, budesonide 0.03–0.04% rinse, or clobetasol 0.05% gel. The rinses were indicated due to the extensive oral surface involved, and the gel was applied in an individual tray to optimize delivery of the steroid to the gums. Both steroid rinses prescribed are not available commercially and indicate that a more potent treatment was necessary relative to commonly used steroid rinses. Interestingly, one of the patients was treated with photobiomodulation, which achieved immediate pain relief. This technique may offer great value for patients in pain who do not benefit from LA or dislike its numbing effect [30,31]. Although the immediate effect may diminish the pain to almost no pain, multiple sessions may be needed while the oral ulcers and erosions gradually heal.

The range of treatments for the salivary gland irAEs included systemic sialogogues, frequent water sipping, over-the-counter moistening agents, gustatory stimulants, salivary gland intraductal irrigation and acupuncture. Of note, the salivary gland intraductal irrigation, which is a minimally invasive procedure previously reported as a simple and safe procedure for relief of dry mouth [32], was tolerated very well and symptom relief was noticed within the next day. This is the first report of the beneficial effect of intraductal irrigation for salivary gland irAEs. Antifungals were prescribed when oral candidiasis was suspected. Most patients responded gradually following the hold of the immunotherapy and administration of systemic steroids; however, some patients only responded partially or did not respond. Given that the dry mouth lasts for at least a few months, measures to prevent dental decay should be put into place, such as motivating the patient to practice meticulous oral hygiene and to use higher-concentration fluoride preparations.

This paper demonstrates the application of common concepts in the management of chronic oral diseases, in the context of immune-mediated oral toxicity. It adds to the current knowledge of the clinical signs and unique treatments that may be offered to patients with oral irAEs. With this, it complements the consensus about common practice for management of toxicities from immunotherapy [33].

## 5. Conclusions

This paper presented a detailed description of oral mucosal irAEs and salivary gland irAEs, and their long-term implications. There is similarity between these oral irAEs and oral manifestations of autoimmune diseases. To the best of our knowledge, this is the first report of oral cancers and PL that developed in patients treated with pembrolizumab or nivolumab, which raises concerns about long-term oral malignant transformation.

## Figures and Tables

**Table 1:** Patient demographics. (**a**) Patients A1–A7; (**b**) Patients A8–C3.

**(a)**							
**Patient #**	**A1**	**A2**	**A3**	**A4**	**A5**	**A6**	**A7**
**Age**	72	62	33	60	52	56	63
**Gender**	M	M	F	M	M	F	M
**Cancer Diagnosis**	Melanoma	Adenocarcinoma of lung	Melanoma	Nasopharyngeal carcinoma	Melanoma	Malignant peripheral nerve sheet tumor of lung	Adenocarcinoma of lung
**Metastasis**	Y	Y	Y	N	Y	Y	Y
**Metastasis to**	Liver, mediastinum	Brain, spinal cord	Lung	-	Lymph nodes	Adrenal, subcutaneous pancreas	Lymph nodes
**Smoking**	N	Y-current	N	N	N	N	N-former
**Alcohol**	N	N	N	N	Occasionally	N	N
**Smoking History**	-	50 years (=100 year-pack)	-	-	-	-	20 years (=20 year-packs)
**Co-morbidities**	None prior to cancer	DM, GERD	None	None	HTN, Hypothyroidism, BPH	GERD	PE
**Medications**	Zoledronic acid, Calcium, Prednisone *, Lenvatinib	Zoledronic acid, Lansoprazole, Amlodipine + Valsartan (Duplex), Simvastatin, Aspirin, Thiazide, Metformin	None	None	Ramipril	Omeprazole	Erdafitinib, Enoxaparin
**Cancer Treatment**	Surgery	RT (not to H&N)	Surgery, RT (not to H&N)	Surgery, RT to H&N, CT	Surgery	CT	RT (not to H&N), CT, bevacizumab
**Immunotherapy Agent**	Nivolumab + Ipilimumab	Nivolumab + Ipilimumab	Nivolumab + Ipilimumab, Nivolumab	Pembrolizumab	Nivolumab	Pembrolizumab	Nivolumab
**Dose**	1 mg/kg × 1/3 wk. + 3 mg/kg × 1/3 wk.	1 mg/kg × 1/3 wk. + 3 mg/kg × 1/3 wk.	1 mg/kg × 1/3 wk. + 3 mg/kg × 1/3 wk; 240 mg × 1/2 wk.	200 mg × 1/3 wk.	240 mg × 1/2 wk.	200 mg × 1/3 wk.	240 mg × 1/2 wk.
**# Doses by the first visit in Oral Medicine**	2	5	1, 4	9	9	3	15
**(b)**							
**Patient #**	**A8**	**B1**	**B2**	**B3**	**C1**	**C2**	**C3**
**Age**	81	52	83	81	79	68	61
**Gender**	M	F	F	M	M	M	M
**Cancer Diagnosis**	Renal cell carcinoma	Melanoma	Cervical	Melanoma	Melanoma, Eye	Esophageal Adenocarcinoma	Tongue SCC
**Metastasis**	Y	Y	Y	Y	Y	N	Y
**Metastasis to**	Skeletal, lungs, prostate	Lung, mediastinal lymph nodes, bone	Lung, peritoneal	Lymph node	Parotid gland	-	Lymph node, scalp
**Smoking**	N	N	N	N	N	N-former	-
**Alcohol**	N	N	N	N	1 glass/day	N	-
**Smoking History**	-	-	-	-	-	2 ppd, 80 pack-years	-
**Co-morbidities**	Osteoarthritis, Renal failure, Hypothyroidism, GERD	DM	DM, HTN, Osteoporosis, Hyperthyroidism, Dyslipidemia, AF	BPH, HTN	Prostate cancer, Sarcoidosis, Chronic kidney disease, Aortic valve disorder, Mitral valve disorder	BPH, HTN, AF, CAP, Urothelial cancer, GERD	Factor VII deficiency, hyperlipidemia, HTN, Hypothyroidism, PE
**Medications**	Zoledronic acid, Prednisone, * Levothyroxine, Atorvastatin, Tamsulosin, Famotidine	Metformin/Sitagliptin, Amitriptyline, Oxycodone + Acetaminophen, Oxycodone + Naloxone, Acetazolamide	Candesartan, Mirabegron, Levothyroxine, Metformin, Atenolol, Amiodarone, Simvastatin, Rivaroxaban	Doxazosin Escitalopram Spironolactone, Zolpidem, Fesoterodine, Valsartan	Prednisone, Nifedipine, Atorvastatin, Vitamin B-12	Omeprazole, Magnesium hydroxide, Tamsulosin	Tramadol, Lorazepam, Aspirin, Metronidazole, Famotidine, Duloxetine
**Cancer Treatment**	Surgery, RT (not to H&N)	CT, RT (not to H&N), Lenvatinib	CT, RT (not to H&N), encorafenib + binimetinib, Lenvatinib	None	Surgery	Surgery, CT, RT to the N	Surgery, CT, RT to H&N, Cetuximab
**Immunotherapy Agent**	Nivolumab + Ipilimumab, Nivolumab	Pembrolizumab	Pembrolizumab	Pembrolizumab	Pembrolizumab	Nivolumab	Pembrolizumab
**Dose**	1 mg/kg × 1/3 wk. + 3 mg/kg × 1/3 wk. for 8 mo; 240 mg × 1/2 wk. for 26 mo.	200 mg × 1/3 wk.	200 mg × 1/3 wk.	200 mg × 1/3 wk.	400 mg × 1/6 wk.	240 mg × 1/2 wk.	200 mg × 1/3 wk.
**# Doses by the first visit in Oral Medicine**	11, 52	22	16	10	5	2	14

BPH—benign prostatic hyperplasia, CAP—community acquired pneumonia, CT—chemotherapy, DM—Diabetes Mellitus, GERD—gastroesophageal reflux disease, H&N—head and neck, HTN—Hypertension, AF –Atrial Fibrillation, PE—pulmonary thromboembolism, PPD—pack per day, RT—radiotherapy, * prednisone was prescribed due to immunotherapy-related arthritis, # number.

**Table 2:** Oral mucosal immunotherapy related adverse effects.

Patient #		A1	A2	A3	A4	A6	A7	A8	B1	B2	B3	C1	C2
Pain Level		0	0	10	5	7	10	0	9	10	10	N/A	7
**Sensitivity Level**		10	0	7	0	0	0	0	9	10	10	N/A	N/A
**Type of symptom**		Burning, difficulty eating, 26 lbs. weight loss		Burning, dysphagia, cannot eat or drink for a week	Pain	Pain, dysphagia	Pain, difficulty eating		Pain, difficulty swallowing, bleeding	Pain, sensitivity to spicy/acidic foods, difficulty swallowing	Pain, burning sensation, difficulty eating and swallowing	Sensitivity to touch/foods, cracked and swollen lips, mouth bleeding	Pain, swelling, taste loss, bleeding, unable to eat, 30 lbs. weight loss
**Onset Time since** **immunotherapy**		11 mo.		12 mo.	5 mo.	2 mo.	12 mo.		2 mo.	10 mo.	8 mo.	2 mo.	2–3 wks.
**Aggravating Factors**				Eating	Salt and sour intake	Salt and sour intake	Eating					RT to the left parotid	RT to esophagus 8 mo. prior
**Type of lesions**													
	**Lichenoid**		x	x	x		x	x	x	x	x	x	x
	**Erythema**	x			x	x	x		x	x	x	x	x
	**Ulcer**			x	x		x		x	x	x	x	x
	**Other**			Lip crusts			Bleeding, Pigmentation		Thinning of the mucosa	Thinning of the mucosa	Thinning of the mucosa	Mucoid saliva, thin white plaque	Mucoid saliva
**Surface involved**													
	**Lips**			x					x	x	x	x	
	**Buccal mucosa**			x	x	x	x	x	x	x		x	x
	**Labial mucosa**	x				x		x	x	x		x	x
	**Tongue Dorsum**	x		x					x		x		
	**Tongue Sides**					x			x			x	x
	**Palate–Hard**	x	x				x					x	x
	**Palate–Soft**										x		
	**Floor of Mouth**										x		
	**Gingival tissue**								x	x	x	x	x
**Microscopic** **assessment**													
	**H&E**	N/A	Mucosal acanthosis, hyperkeratosis, and mild neutrophilic infiltration.	Lichenoid reaction with ulceration (no dysplasia)	Lichen planus pemphigoides	N/A	Lichenoid reaction (no dysplasia)	N/A	Subepithelial split	N/A	Peripheral giant cell granuloma	Marked mixed inflammation involving squamous mucosa	Ulcerated and markedly inflamed squamous mucosa
													-squamous mucosa with lichenoid pattern esophagitis
	**DIF**	N/A	IgA, IgG, IgM, C3, Fibrinogen—negative	N/A	IgA, IgG, Fibrinogen—intercellular surface staining IgM, C3—weak intercellular surface staining	N/A	IgA, IgG, IgM, C3—no staining Fibrinogen—linear basement membrane zone	N/A	IgA—+/- junction IgG—positive +3 junction C3—positive +2 junction IgM, Fibrinogen—negative	N/A	N/A	N/A	IgG, IgA, IgM, C3, Fibrinogen–negative

DIF—direct immunofluorescence, H&E—hematoxylin and eosin, Mo.—months, RT—radiotherapy, N/A—not available, wks.—week. # number.

**Table 3:** Treatment for the oral mucosal immunotherapy related adverse effects.

Patient #	A1	A3	A4	A6	A7	A8	B1	B2	B3	C1	C2
**Immunotherapy reduced/held temporarily/stopped**	No *	Stopped	No	No	No	No	Stopped	Stopped	Stopped	Stopped	Stopped
**Treatment** **(systemic)**	None	Prednisone 50 mg	None	None	None	None	Prednisone 40 mg	None	None	Prednisone 60 mg	Prednisone 40 mg; Clotrimazole; Fluconazole; Nystatin
**Treatment** **(topical)**	Nystatin	Dexa. 0.05% mouthwash	None	Bud. 0.025% mouthwash; PBM	Dexa. 0.04% mouthwash; Benzydamine mouthwash 0.15%	None	Dexa. 0.05%. mouthwash; later, clobetasol in individual tray	Dexa. 0.05% mouthwash, reduced to 0.02% due to adverse effects	Dexa. 0.05% mouthwash	Nystatin, CHX, moistening agents	Diphenhydramine–Lidocaine–Maalox compound, Benzocaine 20% gel, Lidocaine 2% rinse, salty water rinse, Hydrogen peroxide rinse, Dexa.-Nystatin-Lidocaine compound, Vaseline
**Response**	Fair	Excellent	Lost to F/U	No response to topical Bud; good response to PBM	No response with Dexa. ** Relief for 30 min with Benzydamine	N/A	Initial good response to systemic prednisone and topical Dexa. Exacerbation following tapering down. Very good response to clobetasol in individual tray	Good response	Good response	Good response-rapid, improvement on systemic prednisone, but slow healing over months	Fair

Dexa.—dexamethasone, Bud.—budesonide, CHX—chlorhexidine, F/U—follow-up, N/A—not applicable, PBM—photobiomodulation * immunotherapy was stopped 5 months prior to the onset of oral irAE due to nausea and vomiting. ** rinse was diluted by the patient to 0.02%. # number.

**Table 4:** Salivary gland immunotherapy related adverse effects.

**Patient #**	**A1**	**A5**	**A6**	**B1**	**B2**	**B3**	**C1**	**C3**
**Dry mouth (0–10)**	7.5	9	10	9	8	4		
**Type of symptoms**	Xerostomia, loss of appetite, waking from sleep	Dryness, cannot tolerate dry intake, waking from sleep because of xerostomia	Severe dryness, dysphagia, difficulty speaking	Severe dryness	Dryness, dysphagia	Dry lips		Dryness, sticky mucous
**Onset time since immunotherapy**	11 mo.	4 mo.	8 mo.	22 mo.	2 mo.	8 mo.	1 mo.	20 mo.
**Aggravating factors**	Mouth breathing	Talking, eating						RT *, medication, mouth breathing
**Signs**	Thick, sticky, frothy saliva	Thick, sticky, frothy saliva, glossy appearance, depapillated tongue, oral candidiasis	Glossy appearance	Glossy appearance; mirror sticks to surface	Glossy appearance, sticky saliva	Dry and cracked lips	Thick, sticky, frothy saliva; dry and cracked lips; mucoid strings; glossy tongue appearance, oral surface is wet	Oral surface is very dry

* Palliative radiotherapy to the neck was delivered following the treatment with immunotherapy and may have increased the tendency for dry mouth. # number.

**Table 5:** Treatment for the salivary gland immunotherapy related adverse effects.

**Patient #**	**A1**	**A5**	**A6**	**B1**	**B2**	**B3**	**C1**
**Immunotherapy reduced/held temporarily/stopped**	No *	No	No	Stopped long before dry mouth	Stopped	Stopped	Stopped
**Treatment** **(systemic)**	None	Pilocarpine 5 mg ×3/d	None	Prednisone 15 mg	None	None	Prednisone 60 mg
**Treatment** **(topical)**	Sipping water, gustatory stimulants, liquid moistening agents	Sipping water with lemon, gustatory stimulants, liquid moistening agents, nystatin	Sipping water, gustatory stimulants, liquid moistening agents	Liquid moistening agent	Dissolving moistening agents	None	Acupuncture, nystatin, CHX, gustatory stimulants, dissolving moistening agents, Salivary gland intraductal irrigations
**Response**	Fair	Fair	Lost to F/U	Partial improvement	No improvement	Gradual spontaneous improvement	Rapid improvement since on prednisone; additional immediate improvement after salivary gland intraductal irrigations.

* Immunotherapy was stopped 5 months prior to the onset of dry mouth. CHX—chlorhexidine, F/U—follow-up; Dissolving moistening agents—over the counter adhesive discs (e.g., Xylimelts). Liquid moistening agents—over the counter preparations (e.g., Biotene). Gustatory stimulants—suck on sugarless, sour–sweet, hard candies. # number.

## Data Availability

The data presented in this study are available in this article.

## References

[1] Brahmer J.R., Lacchetti C., Thompson J.A. (2018). Management of Immune-Related Adverse Events in Patients Treated with Immune Checkpoint Inhibitor Therapy: American Society of Clinical Oncology Clinical Practice Guideline Summary. J. Oncol. Pract..

[2] Kennedy L.B., Salama A.K.S. (2020). A review of cancer immunotherapy toxicity. CA Cancer J. Clin..

[3] Lee D.J., Lee H.J., Farmer J.R., Reynolds K.L. (2021). Mechanisms Driving Immune-Related Adverse Events in Cancer Patients Treated with Immune Checkpoint Inhibitors. Curr. Cardiol. Rep..

[4] Chen T.W., Razak A.R., Bedard P.L., Siu L.L., Hansen A.R. (2015). A systematic review of immune-related adverse event reporting in clinical trials of immune checkpoint inhibitors. Ann. Oncol..

[5] Martins F., Sofiya L., Sykiotis G.P., Lamine F., Maillard M., Fraga M., Shabafrouz K., Ribi C., Cairoli A., Guex-Crosier Y. (2019). Adverse effects of immune-checkpoint inhibitors: Epidemiology, management and surveillance. Nat. Rev. Clin. Oncol..

[6] Ramos-Casals M., Brahmer J.R., Callahan M.K., Flores-Chavez A., Keegan N., Khamashta M.A., Lambotte O., Mariette X., Prat A., Suarez-Almazor M.E. (2020). Immune-related adverse events of checkpoint inhibitors. Nat. Rev. Dis. Prim..

[7] Klein B.A., Alves F.A., de Santana Rodrigues Velho J., Vacharotayangul P., Hanna G.J., LeBoeuf N.R., Shazib M.A., Villa A., Woo S.B., Sroussi H. (2022). Oral manifestations of immune-related adverse events in cancer patients treated with immune checkpoint inhibitors. Oral Dis..

[8] Bustillos H., Indorf A., Alwan L., Thompson J., Jung L. (2022). Xerostomia: An immunotherapy-related adverse effect in cancer patients. Support. Care Cancer.

[9] Amy D.P.B., Shalabi A., Finfter O., Birenzweig Y., Zadik Y. (2020). Severe chronic nonlichenoid oral mucositis in pembrolizumab-treated patients: New cases and a review of the literature. Immunotherapy.

[10] Elad S., Yarom N., Zadik Y., Kuten-Shorrer M., Sonis S.T. (2022). The broadening scope of oral mucositis and oral ulcerative mucosal toxicities of anticancer therapies. CA Cancer J. Clin..

[11] Jacob J.S., Dutra B.E., Garcia-Rodriguez V., Panneerselvam K., Abraham F.O., Zou F., Ma W., Grivas P., Thompson J.A., Altan M. (2021). Clinical Characteristics and Outcomes of Oral Mucositis Associated with Immune Checkpoint Inhibitors in Patients with Cancer. J. Natl. Compr. Cancer Netw..

[12] Decaux J., Magremanne M. (2020). Medication-related osteonecrosis of the jaw related to epacadostat and pembrolizumab. J. Stomatol. Oral Maxillofac. Surg..

[13] Warner B.M., Baer A.N., Lipson E.J., Allen C., Hinrichs C., Rajan A., Pelayo E., Beach M., Gulley J.L., Madan R.A. (2019). Sicca Syndrome Associated with Immune Checkpoint Inhibitor Therapy. Oncologist.

[14] Xu Y., Wen N., Sonis S.T., Villa A. (2021). Oral side effects of immune checkpoint inhibitor therapy (ICIT): An analysis of 4683 patients receiving ICIT for malignancies at Massachusetts General Hospital, Brigham & Women’s Hospital, and the Dana-Farber Cancer Institute, 2011 to 2019. Cancer.

[15] Yura Y., Hamada M. (2022). Oral Immune-Related Adverse Events Caused by Immune Checkpoint Inhibitors: Salivary Gland Dysfunction and Mucosal Diseases. Cancers.

[16] Ioannides D., Vakirlis E., Kemeny L., Marinovic B., Massone C., Murphy R., Nast A., Ronnevig J., Ruzicka T., Cooper S.M. (2020). European S1 guidelines on the management of lichen planus: A cooperation of the European Dermatology Forum with the European Academy of Dermatology and Venereology. J. Eur. Acad. Dermatol. Venereol..

[17] Cheng Y.S., Gould A., Kurago Z., Fantasia J., Muller S. (2016). Diagnosis of oral lichen planus: A position paper of the American Academy of Oral and Maxillofacial Pathology. Oral Surg. Oral Med. Oral Pathol. Oral Radiol..

[18] Duan S., Zhang X., Wang F., Shi Y., Wang J., Zeng X. (2021). Coexistence of oral mucous membrane pemphigoid and lichenoid drug reaction: A case of toripalimab-triggered and pembrolizumab-aggravated oral adverse events. Oral Surg. Oral Med. Oral Pathol. Oral Radiol..

[19] Abdalla-Aslan R., Keshet N., Zadik Y., Aframian D.J., Nadler C. (2021). Standardization of terminology, imaging features, and interpretation of CBCT sialography of major salivary glands: A clinical review. Quintessence Int..

[20] US Department of Health and Human Services, National Institutes of Health, National Cancer Institute (2017). Common Terminology Criteria for Adverse Events (CTCAE) Version 5.0. https://ctep.cancer.gov/protocoldevelopment/electronic_applications/docs/CTCAE_v5_Quick_Reference_8.5x11.pdf.

[21] Harris J.A., Huang K., Miloslavsky E., Hanna G.J. (2022). Sicca syndrome associated with immune checkpoint inhibitor therapy. Oral Dis..

[22] Hakeem A., Bhattacharyya I., Aljabri M., Bindakhil M., Pachigar K., Islam M.N., Cohen D.M., Fitzpatrick S.G. (2019). Lichenoid reaction with granulomatous stomatitis: A retrospective histologic study of 47 patients. J. Oral Pathol. Med..

[23] James D.G. (2000). A clinicopathological classification of granulomatous disorders. Postgrad. Med. J..

[24] Atsuta Y., Suzuki R., Yamashita T., Fukuda T., Miyamura K., Taniguchi S., Iida H., Uchida T., Ikegame K., Takahashi S. (2014). Continuing increased risk of oral/esophageal cancer after allogeneic hematopoietic stem cell transplantation in adults in association with chronic graft-versus-host disease. Ann. Oncol..

[25] Warnakulasuriya S., Kujan O., Aguirre-Urizar J.M., Bagan J.V., Gonzalez-Moles M.A., Kerr A.R., Lodi G., Mello F.W., Monteiro L., Ogden G.R. (2021). Oral potentially malignant disorders: A consensus report from an international seminar on nomenclature and classification, convened by the WHO Collaborating Centre for Oral Cancer. Oral Dis..

[26] Iocca O., Sollecito T.P., Alawi F., Weinstein G.S., Newman J.G., De Virgilio A., Di Maio P., Spriano G., Pardinas Lopez S., Shanti R.M. (2020). Potentially malignant disorders of the oral cavity and oral dysplasia: A systematic review and meta-analysis of malignant transformation rate by subtype. Head Neck.

[27] Buchbinder E.I., Desai A. (2016). CTLA-4 and PD-1 Pathways: Similarities, Differences, and Implications of Their Inhibition. Am. J. Clin. Oncol..

[28] Villa A., Kuten-Shorrer M. (2023). Pathogenesis of Oral Toxicities Associated with Targeted Therapy and Immunotherapy. Int. J. Mol. Sci..

[29] El-Howati A., Thornhill M.H., Colley H.E., Murdoch C. (2023). Immune mechanisms in oral lichen planus. Oral Dis..

[30] Finfter O., Avni B., Grisariu S., Haviv Y., Nadler C., Rimon O., Zadik Y. (2021). Photobiomodulation (low-level laser) therapy for immediate pain relief of persistent oral ulcers in chronic graft-versus-host disease. Support. Care Cancer.

[31] Finfter O., Cohen R., Hanut A., Gavish L., Zadik Y. (2023). High-power laser photobiomodulation therapy for immediate pain relief of refractory oral mucositis. Oral Dis..

[32] Aframian D.J., Baaton S., Mazor S., Nadler C., Keshet N., Haviv Y., Zadik Y., Schwimmer-Noy R., Shay B., Almoznino G. (2019). Improvement of dry mouth following intraductal irrigation of salivary glands. Oral Dis..

[33] Haanen J., Obeid M., Spain L., Carbonnel F., Wang Y., Robert C., Lyon A.R., Wick W., Kostine M., Peters S. (2022). Management of toxicities from immunotherapy: ESMO Clinical Practice Guideline for diagnosis, treatment and follow-up. Ann. Oncol..

